# *Lactobacillus*-driven feed fermentation regulates microbiota metabolism and reduces odor emission from the feces of pigs

**DOI:** 10.1128/msystems.00988-23

**Published:** 2023-11-30

**Authors:** Dongyan Zhang, Haifeng Ji, Sixin Wang, Yajuan Liu, Meixia Chen, Hui Liu

**Affiliations:** 1 Institute of Animal Science and Veterinary Medicine, Beijing Academy of Agriculture and Forestry Sciences, Beijing, China; 2 Mountainous Area Research Institute of Hebei Province, Hebei Agricultural University, Baoding, China; Iowa State University, Ames, Iowa, USA

**Keywords:** *Lactobacillus *fermentation, pig farming, odor emission, fecal storage, microbiota and metabolites

## Abstract

Fermentation by *Lactobacillus* can increase the nutritional value of feed, which is advantageous for pig production. However, the effects of *Lactobacillus*-driven feed fermentation on the emission of odors from pig feces are unclear. Here, we evaluated how feed fermentation by *Lactobacillus* affects the concentration of odor molecules in feces (CO_2_, NH_3_, H_2_S, indole, and skatole) and analyzed the changes in fecal microbiota and metabolites during fecal storage. Pigs reared on fermented feed had a higher average daily gain (ADG) and lower feed-to-gain (*F*/*G*) ratio than the control group. By the 3rd and 5th days, the concentration of CO_2_ in feces from pigs in the fermentation group significantly decreased, and the contents of H_2_S, NH_3_, and skatole in the fermentation group were significantly lower than those in the control group on the 1st, 3rd, and 5th days of fecal storage. The fermentation group exhibited a higher Bacteroidetes abundance on the 3rd and 5th days. With prolonged fecal storage, the fermentation group exhibited a higher abundance of *Weissella* and *Lactobacillus* and a lower abundance of *Streptococcus*. The *Lactobacillus* fermentation group also showed lower levels of secondary amino acid metabolites, such as 6-hydroxyhexanoic acid and 5-aminopentanoic acid. The pure strain *Weissella cibaria* ZWC030, which could inhibit the production of skatole *in vitro*, was successfully isolated from the fermentation group. Overall, the study revealed the interrelationships among fecal microbiota, metabolites, and odor molecules. The findings provide a new avenue for the application of *Lactobacillus*-driven feed fermentation to promote ADG, decrease the *F*/*G* ratio in pigs during growth, and reduce environmental pollution.

**IMPORTANCE:**

Our present study showed that dietary supplementation with feed fermented by *Lactobacillus* could promote the growth performance of pigs, regulate the microbiota, and inhibit the growth of harmful bacteria. It could prevent the accumulation of toxic substances and reduce odor emission from pig feces, thereby reducing environmental pollution. In addition, one key triumph of the present study was the isolation of *Weissella cibaria* ZWC030, and the strain could inhibit the production of skatole *in vitro* in our present results.

## INTRODUCTION

Concerns regarding the emission of greenhouse gases from livestock respiration and manure are a global problem (1, 2). These odorous compounds emitted from feces not only reduce the production performance of livestock (3, 4) but also put farm workers and people living in nearby areas at risk of respiratory distress and illnesses (5, 6). Furthermore, these high odorous emissions can also cause serious ecological disturbances, inducing phenomena such as acid rain and nitrification (7, 8).

Many studies have suggested that nutrient digestibility is tightly correlated with odor emissions (9). When the levels of fermentable carbohydrates in the feed are low, the gut microbiota becomes more adept at fermenting proteins (10). NH_3_ is the main product of protein fermentation in the intestine (11). Meanwhile, H_2_S is a volatile generated by the microbial degradation of sulfur-containing amino acids (12, 13), and skatole is generated via the anaerobic bacterial transformation of tryptophan (14). It has been demonstrated that reductions in dietary protein can lower the emission of NH_3_ (15, 16). Previous studies have used feed additives such as benzoic acid (17), sodium butyrate (18), and xylanase enzymes (19) to reduce the emission of odors from manure. All the mechanisms regulating odor production have been linked to the composition of gut microbes (20–22). Therefore, regulating the composition of intestinal microbes, their metabolic activity, and the intestinal environment may represent a feasible strategy for reducing the production of fecal odors at their source.

All species of *Lactobacillus*, a ubiquitous member of the intestinal microbiota, can reduce pH by producing lactic acid as the end product of carbohydrate fermentation. Some *Lactobacillus* strains also generate anti-microbial peptides and other metabolites that can suppress the growth of pathogenic bacteria (23–25). During *Lactobacillus* fermentation, cell wall components, phytates, and other anti-nutritional factors present in grains can be partially degraded, and the nutritional content of feed ingredients can thus be enhanced by freeing encapsulated nutrients and making them available for livestock, such as pigs (26). In addition, some metabolites produced following fermentation, including lactic acid, can inhibit pathogen multiplication and increase short-chain fatty acid production in the gut by reducing intestinal pH (27, 28). In weaned pigs fed *Lactobacillus*-based probiotics, the resultant increase in nutrient digestibility leads to a decrease in the amount of substrate available for microbial fermentation in the colon, reducing the emission of ammonia (NH_3_), hydrogen sulfide (H_2_S), and total mercaptans (20). However, the changes in fecal odor, microbial composition, and metabolites after supplementation are still unclear.

A previous study suggested that fermentation with *Lactobacillus* can enhance the nutritional content of wheat bran and improve the growth performance of pigs, while also decreasing the emission of heavy metals (29). We hypothesized that after supplementation with fermented feed, the changes in the microbiota could influence fecal metabolism, and these changes may promote decreases in odor emission. Nevertheless, the mechanism underlying this possible link remained to be established.

Here, we tested the effects of feed fermented by *Lactobacillus acidophilus* ZLA012 (*L. acidophilus* ZLA012), a strain isolated from a healthy growing pig, on the concentration of the odor components carbon dioxide (CO_2_), NH_3_, H_2_S, indole, and skatole in pig feces. Further, we sought to assess how supplementation with fermented feed affects changes in the abundance of fecal microbiota and metabolites with prolonged fecal storage. In addition, we isolated a *Weissella* strain highly related to odor emission and studied its influence on the degradation of skatole *in vitro*.

## MATERIALS AND METHODS

### Bacterial strain

We previously isolated *L. acidophilus* ZLA012 from the feces of a healthy growing pig and stored it at the China General Microbiological Culture Collection Center (preservation no.: CGMCC 8491). *L. acidophilus* ZLA012 was cultured in de Mann, Rogosa, and Sharpe (MRS) medium. Cultures were centrifuged to harvest cells, which were then re-suspended in protectant and freeze-dried (Epsilon 2-60; Martin Christ GmbH, Germany) to generate a powder (10^10^ CFU/g).

### Feed fermentation

Feed was solid fermented in a clean plastic bucket with screw-sealing covers. The optimum fermentation conditions were as follows: *L. acidophilus* ZLA012 concentration of 0.35% (wt/wt), feed-to-water ratio of 1:1.2, fermentation time of 24 h, and fermentation temperature of 37°C. The viable count of the fermented feed was 5.75 × 10^9^ CFU/g, and the final pH was 4.11.

### Animal experiments

All animal experiments were conducted at a commercial pig farm (Beijing). Seventy-two male and female landrace × large white crossbred pigs (100 days of age) with comparable initial body weights (33.85 ± 3.31 kg) were selected and separated into weight- and sex-matched groups. There were a total of two groups, each with six replicate pens and six pigs/pen. Pigs in the control group were reared on a basal diet, whereas pigs in the fermentation group were reared on a basal diet supplemented with 1% fermented feed. Table 1 shows the composition and nutrient content of the basal feed, which met the requirements of the Chinese standard GB/T 39235-2020—Pig Feed Standard(30) for growing pigs. The pigs were maintained in 12 adjacent enclosures on a concrete floor in a controlled environment. Throughout the 30 days of the experiment, the pigs were granted free access to one of two dry diets and water from one feeder and one nipple drinker. Basal diets were formulated every 5 days, and the fermented feed was added to the basal diet and mixed well before feeding each day. Both of the feed used the same feeding methods.

**TABLE 1:** Composition of the basal feed of growing pigs (as fed-basis)

Ingredients (%)	Contents

Maize	68
Soybean meal	24
Wheat bran	4
Premix^*a*^	4
Chemical composition	
Digestible energy^*b*^, MJ/kg	13.46
Crude protein^*c*^, %	16.70
Lysine^*b*^, g/kg	8.50
Methionine^*b*^, g/kg	2.60
Calcium^*c*^, g/kg	6.03
Total phosphorus^*c*^, g/kg	5.21

^*a*^ Each kilogram of complete feed contained the following: vitamin A, 5,512 IU; vitamin D_3_, 2,250 IU; vitamin E, 44 mg; menadione, 3.15 mg; vitamin B_1_, 8.1 mg; vitamin B_2_, 6.4 mg; vitamin B_6_, 3.0 mg; vitamin B_12_, 0.025 mg; niacin, 25 mg; pantothenic acid, 10.8 mg; biotin, 0.12 mg; Mn, 10 mg; Fe, 100 mg; Zn, 100 mg; Cu, 12 mg; I, 0.50 mg; and Se, 0.20 mg.

^*b*^ Calculated nutrient levels.

^*c*^ Measured nutrient levels.

### Growth in pigs

Individual pigs were weighed on day 0 and day 30. The average daily gain (ADG) was calculated by total body weight gains (final weight minus initial weight) during measure periods divided by the corresponding feeding days. Feed intake per pen based on the pig’s intake of feed was recorded weekly to calculate average daily feed intake (ADFI) and feed-to-gain ratio [*F*/*G*, calculated as (kg feed)/(kg ADG)].

### Collection of fecal samples

Six fecal samples were randomly collected from each group on the final day of the experimental period (day 30), divided into three portions each, and transferred to sterile containers. The samples were frozen in liquid nitrogen and stored at −80°C before being used for additional tests. The odor concentration, microbial composition, and metabolite profile of these samples were analyzed.

### Nitrogen migration test

First, 20 mL of 10% hydrochloric acid solution was mixed well with 200 g of feces. The mixture was subsequently dried at 65°C before being pulverized into small particles and passed through a 40-mesh sieve. The total nitrogen (total N), ammonium nitrogen (NH_4_^+^-N), amide nitrogen (NO_2_^−^-N), and nitrate nitrogen (NO_3_^−^-N) contents of the samples were examined according to standard procedures (NY/T 1116-2014) (31).

### Analysis of odor contents during fecal storage

After fresh feces were collected, 50 g of fecal samples was weighed out and placed into an airtight glass bottle. The gases emitted from the feces were pumped into a circular absorption cell, and the odor molecules were measured using sensors for CO_2_ (SprintIR-WX-60 sensor, 0–2,000 ppm, Gas Sensing Solutions, UK), H_2_S (CLE-0112-400, 0–100 ppm, Honeywell Analytics, Illinois, USA), and NH_3_ (CLE-1052-400, 0–500 ppm, Honeywell Analytics).

The contents of indole and skatole in the pig fecal samples were determined using liquid chromatography (1260 series, Agilent Technologies, Delaware, USA) based on a modified version of the protocol provided by Dehnhard et al. (32). The indole and skatole standards (Sigma-Aldrich, St Louis, MO, USA) were of chromatography grade with a purity >99%. To correct for any losses during the experimental procedure, 2-methylindole was applied as the internal standard. Methanol was used for sample extraction, and samples were further purified on an Amberlite XAD-8 column (Sigma-Aldrich) before high-performance liquid chromatography analysis using UV spectrophotometry at 280 nm (detection limit = 2.5 ng per injection [50 µL], corresponding to 0.2 µg/g feces). The emission of the odor gases CO_2_, H_2_S, NH_3_, indole, and skatole was measured on days 1, 3, and 5 of fecal storage.

### 16S rRNA sequencing

Fecal samples were subjected to DNA extraction using the E.Z.N.A. Stool DNA kit (Omega Bio-tek, GA, USA). The concentration and purity of the DNA were detected after purification. Total DNA isolated from the fecal samples was used as a template to amplify the bacterial 16S rRNA V3–V4 regions using PCR. Bacterial universal primers—338F (5′-ACTC CTACGGGAGGCAGCA-3′) and 806R (5′-GGACTACHVGGGTWTCTAAT-3′)—were used as the forward and reverse primers, respectively. The contents of the reaction mixture were as follows: 5× FastPfu buffer (4 µL), 2.5 mmol/L deoxynucleotide triphosphates (2 µL), 5 µmol/L forward and reverse primers (0.8 µL each), FastPfu polymerase (0.4 µL), DNA template (10 ng), and ddH_2_O (added to make up a volume of 20 µL). The PCR parameters were as follows: 95°C for 3 min; 27 cycles of 95°C for 30 s, 55°C for 30 s, and 72°C for 45 s; and 72°C for 10 min. PCR products obtained from the same sample were mixed and subjected to 2% agarose gel electrophoresis. The amplified products were recovered from the gel and sequenced using the Illumina Miseq PE300 platform. After quality control filtration, the alpha diversity (Chao 1 index, ACE index, Simpson index, and Shannon index) and microbial composition of each sample were determined based on the barcode sequences of each sample. Furthermore, using the RDP classifier Bayesian algorithm, taxonomic analysis was conducted based on a 97% similarity threshold for operational taxonomic units (OTUs), and the bacterial composition was determined across various levels.

### Metabolic profiling

For these analyses, 100-µL aliquots of thawed fecal samples were obtained. The test was conducted using a Waters 2D UPLC (Waters, USA) series Q Exactive high-resolution mass spectrometer (Thermo Fisher Scientific, USA). Metabolite detection was performed using liquid chromatography-tandem mass spectrometry (LC–MS/MS) and Compound Discoverer 3.0 software for LC–MS/MS data processing (Thermo Fisher Scientific, USA). Differential metabolites were screened and further analyzed based on the differential weight contribution values (variable importance in projection[VIP]) of the first two principal components of the partial least squares discriminant analysis (PLS-DA) model and the fold change values.

### Isolation of *Weissella* and inhibition of skatole *in vitro*

In our laboratory, using NH_4_Cl as the only nitrogen source, the *Weissella cibaria* strain ZWC030 was isolated from pig feces through the serial dilution method. The strain was isolated and cultured in MRS medium at 37°C. It was then preserved in 30% glycerol (vol/vol) at −80°C.

First, 250 µmol/L L-tryptophan was added to fecal samples and used for the skatole inhibition test *in vitro. Weissella cibaria* ZWC030 was grown in MRS broth at 37°C overnight. After centrifugation at 6,738 × *g* for 10 min, the supernatant of the culture was collected and treated with protease K at 80°C, pH = 7.0, and pH = 4.5. The supernatant was inoculated into pig feces containing L-tryptophan and stored at 25°C for 8 h and 24 h. The contents of indole and skatole were subsequently determined using the previously described method (see “Analysis of odor contents during fecal storage”).

### Statistical analyses

The general linear model program in SAS software (SAS Institute, Cary, NC, USA) was applied for the analysis of the alpha-diversity indexes and odor composition of the fecal samples. Metabolic profiles and odor contents were analyzed using Tukey’s univariate analysis test, and *P* < 0.05 was considered significant.

## RESULTS

### Pig growth

The relationship between the type of feed and diet, and the values of ADG, ADFI, and *F*/*G* are detailed in Table 2. Compared with the pigs receiving standard feed, the pigs receiving fermented feed had higher final weights and ADG values (*P* = 0.034 and 0.021, respectively). Moreover, they showed lower *F*/*G* ratios (*P* = 0.024).

**TABLE 2:** Effect of fermented feeds on growth indices in pigs

Items	Control group	Fermentation group	*P* value	SEM^*a*^
Initial weight/kg	32.77	32.93	0.125	2.574
Final weight/kg	53.88	55.11	0.034	1.722
ADG, g/day	706.11	739.33	0.021	14.231
ADFI, kg/day	1.592	1.597	0.085	0.024
*F*/*G*	2.25	2.15	0.024	0.047

^*a*^ SEM = standard error of the mean.

### Nitrogen migration test

**Fig 1:**
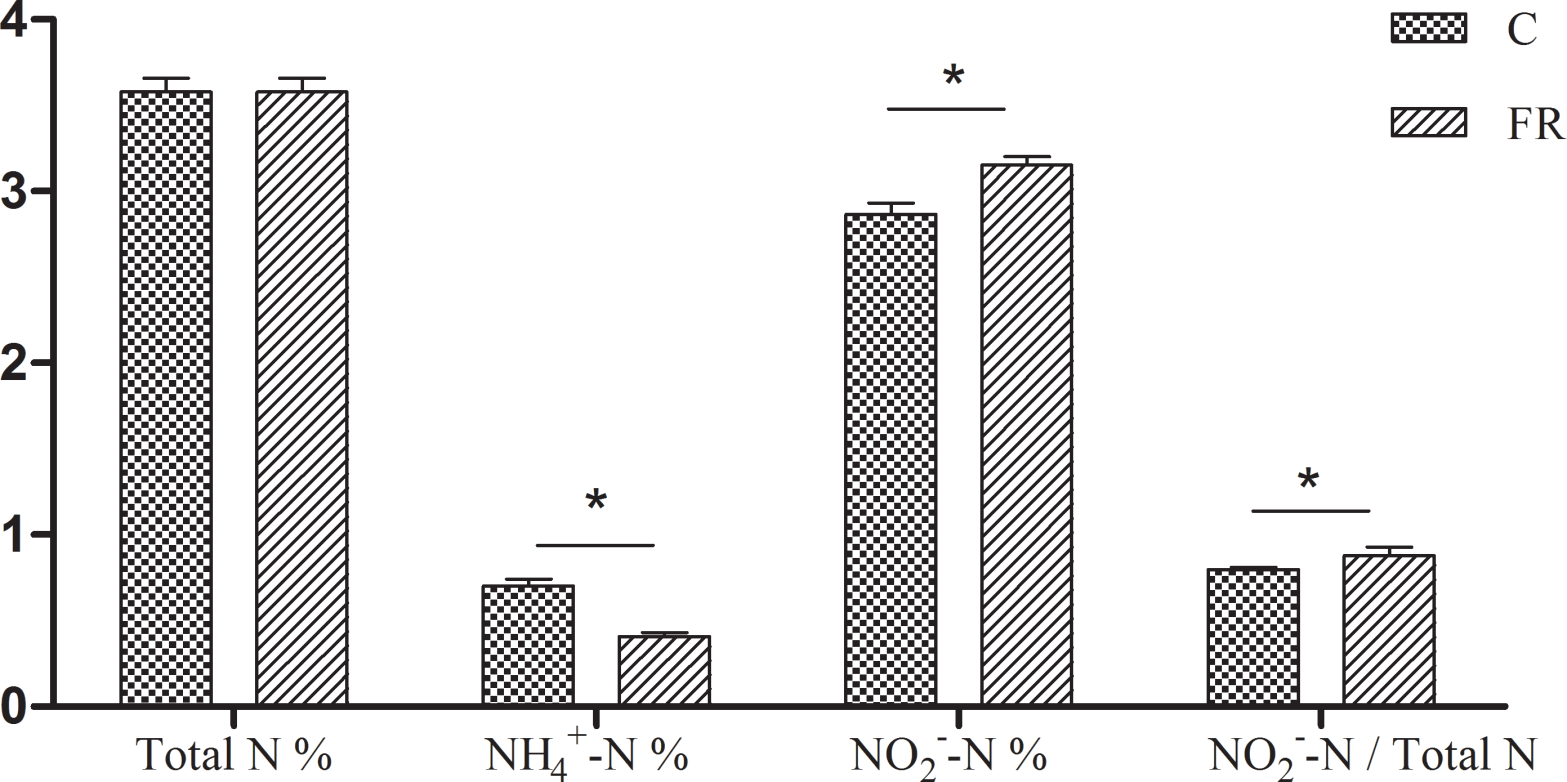
Nitrogen migration test in the control (C) and fermentation (FR) groups. *P < 0.05.

The results showed that the NH_4_^+^-N content was lower and the NO_2_^−^-N content was higher in the fermentation group than in controls. The conversion rates of NO_2_^−^-N/total N in the control and fermentation groups were 80.4% and 88.2%, respectively. This indicated that *Lactobacillus*-driven feed fermentation could reduce nitrogen and ammonia emissions in the environment (Fig. 1).

### Odor content analysis during fecal storage

**Fig 2:**
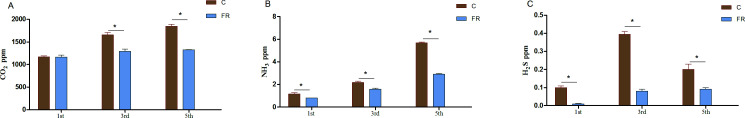
Contents of CO_2_ (A), NH_3_ (B), and H_2_S (C) during the storage of pig feces. C: control group and FR: fermentation group. *P < 0.05.

The CO_2_ emission in this group was significantly decreased than the control group on the 3rd day (*P* = 0.003) and 5th day (*P* = 0.003). The fermentation group showed significantly lower reductions in NH_3_ (*P* = 0.005, 0.001, and 0.002, respectively) and H_2_S (*P* = 0.005, 0.003, and 0.004, respectively) levels on the 1st, 3rd, and 5th days of fecal storage, and the fermentation group exhibited the most obvious reduction in H_2_S emission (Fig. 2).

**Fig 3:**
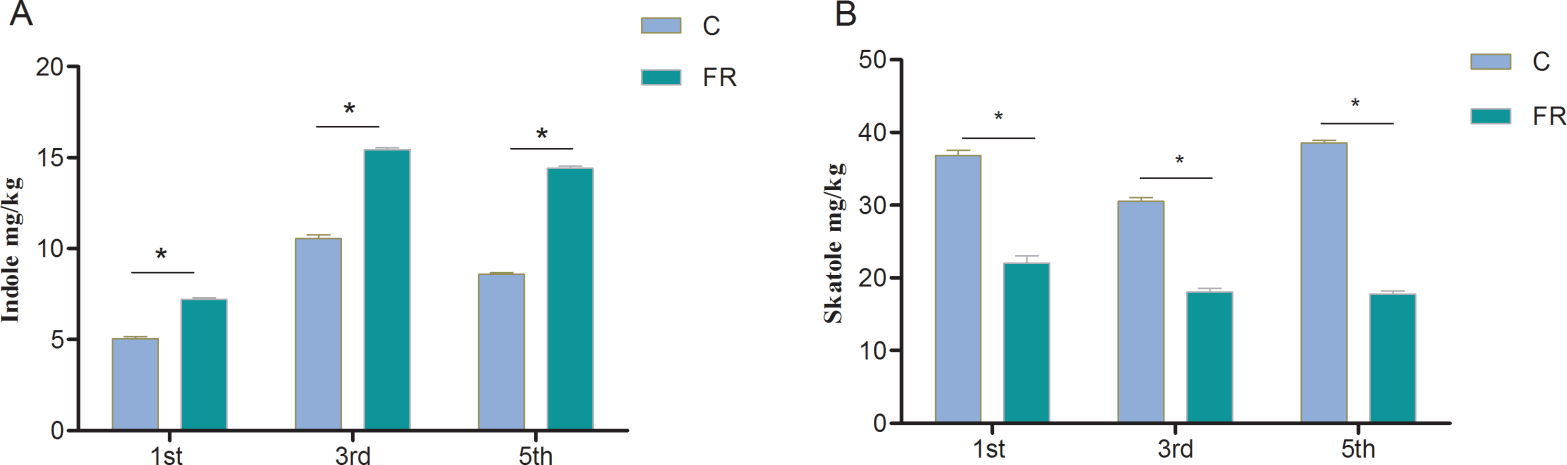
Contents of indole (A) and skatole (B) during the storage of pig feces. C: control group and FR: fermentation group. *P < 0.05.

The indole contents of fecal samples in the group of growing pigs receiving fermented feed were higher than in control group. However, the contents of skatole on the 1st, 3rd, and 5th days of fecal storage were significantly lower than those in the control group (*P* = 0.007, 0.004, and 0.001, respectively) (Fig. 3).

### Alpha-diversity analysis

**Fig 4:**
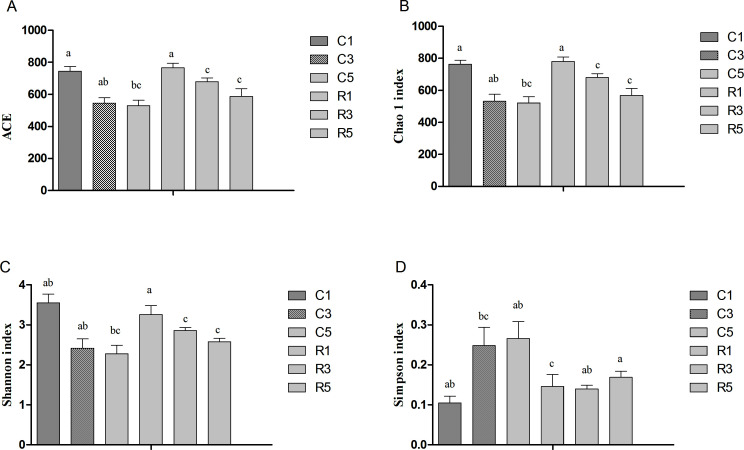
Analysis of the alpha diversity of the fecal microbiota. Indices for bacteria are as follows: (A) Abundance-based coverage estimator (ACE); (B) Chao 1; (C) Shannon; and (D) Simpson. C1: control group on the 1st day; C3: control group on the 3rd day; C5: control group on the 5th day; R1: fermentation group on the 1st day; R3: fermentation group on the 3rd day; and R5: fermentation group on the 5th day. *P < 0.05.

Analyses of ACE and Chao 1 revealed significant decreases in species richness on the 1st, 3rd, and 5th days in both the control group and the fermentation group. Further, based on the ACE, Chao 1, and Shannon indices, we concluded that on the 3rd and 5th days, species richness and diversity were higher in samples from the fermentation group than in controls (Fig. 4).

### Microbial composition of pig feces

**Fig 5:**
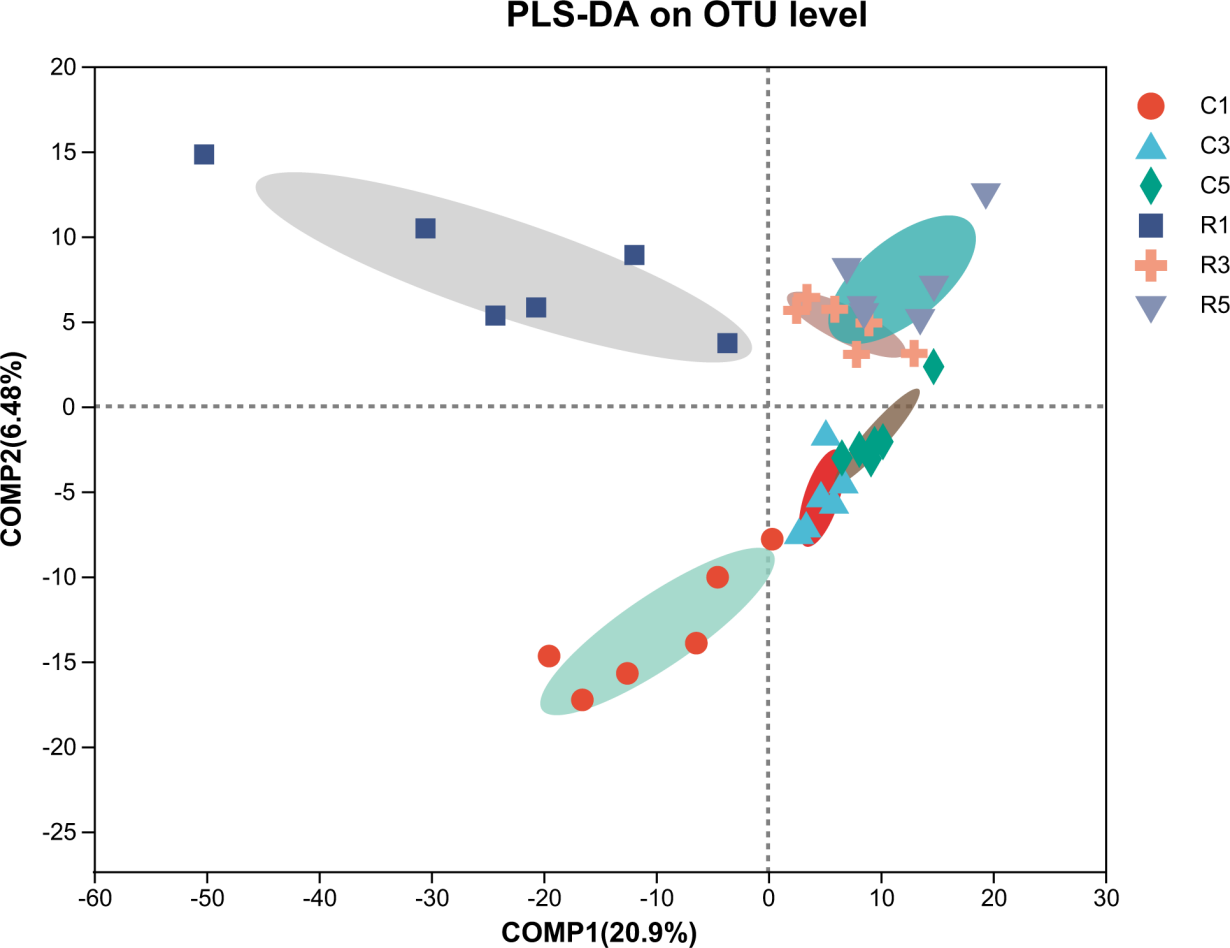
PLS-DA analysis indicating the similarities and differences in OTUs at multiple levels between pigs in two feed groups. C1: control group on the 1st day; C3: control group on the 3rd day; C5: control group on the 5th day; R1: fermentation group on the 1st day; R3: fermentation group on the 3rd day; and R5: fermentation group on the 5th day.

OTU analyses performed using PLS-DA revealed some similarities and differences in the fecal microbiota between the two treatment conditions (Fig. 5). Fecal samples from the fermentation and control groups showed significantly distinct microbial profiles on the 1st, 3rd, and 5th days. However, the microbial profiles of fecal samples on days 3 and 5 were closely related in both treatment groups.

**Fig 6:**
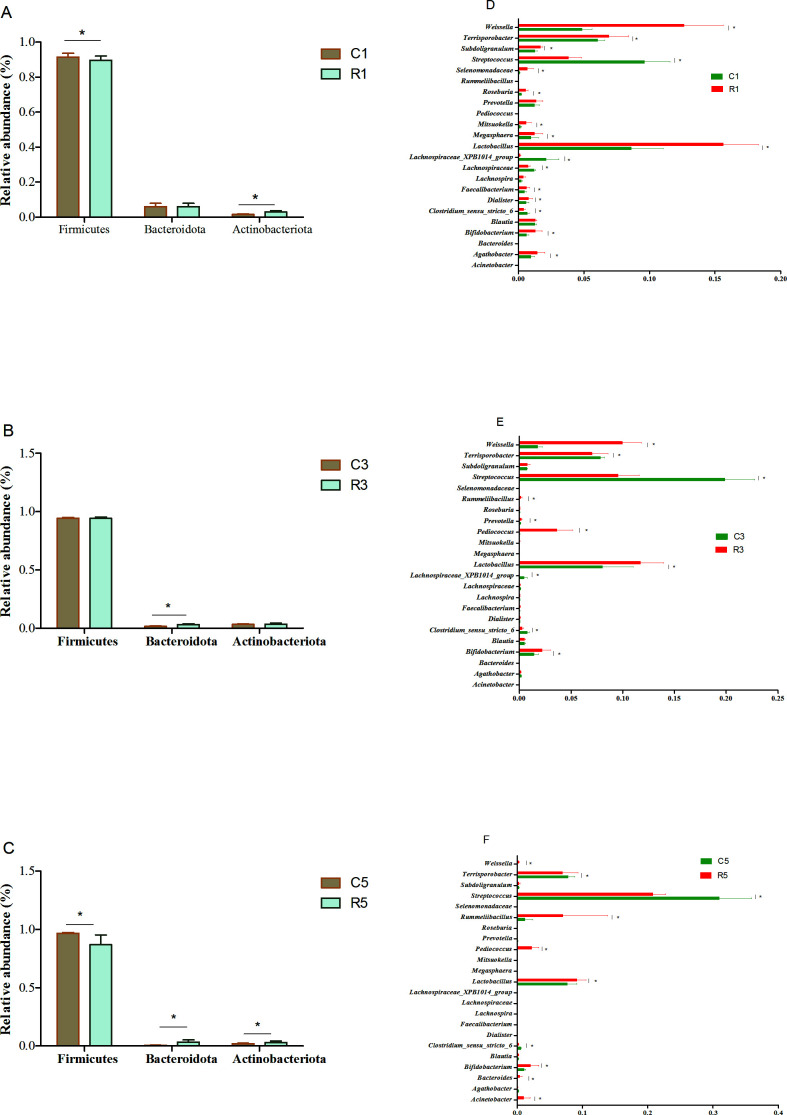
Differences between the microbial composition of fecal samples from pigs. (A, B, C) Differences in the composition of the microbiota were assessed at the phylum level; (D, E, F) Differences in the composition of the microbiota were assessed at the genus level. C1: control group on the 1st day; C3: control group on the 3rd day; C5: control group on the 5th day; R1: fermentation group on the 1st day; R3: fermentation group on the 3rd day; and R5: fermentation group on the 5th day. *P < 0.05.

The abundance of phylum Firmicutes was lower in fecal samples from the fermentation group than in control samples on the 1st (89.72% vs 91.49%) and 5th (87.00% vs 96.69%) days (*P* = 0.034 and 0.027, respectively). However, this value was comparable between the two groups on the 3rd (94.28% vs 94.25%) day of storage (*P* = 0.057). Although the abundance of Bacteroidetes decreased as the storage time increased, it was higher in the fermentation group than in the control group on the 3rd (3.15% vs 1.63%) and 5th (3.26% vs 0.65%) days of storage (*P* = 0.012 and 0.023, respectively). The fermentation group also showed a higher abundance of Actinobacteriota than the control group on the 1st (2.99% vs 1.54%) and 5th (3.01% vs 2.09%) days of storage (*P* = 0.031 and 0.018, respectively) (Fig. 6).

At the genus level, the abundance of most microbes tended to decrease with a prolongation of the fecal storage period with the abundance being lower on the 5th day than on the 1st day. Accordingly, the abundance of the *Weissella* and *Lactobacillus* genera decreased with the storage period. In contrast, *Streptococcus* showed an increase in abundance as the storage time increased. In comparison to the control group, on days 1, 3, and 5 of fecal storage, the fermentation group exhibited significantly higher abundances of *Weissella* (*P* = 0.031, 0.001, and 0.021, respectively) and *Lactobacillus* (*P* = 0.030, 0.034, and 0.046, respectively), and a significantly lower abundance of *Streptococcus* (*P* = 0.024, 0.015, and 0.038, respectively). In line with these findings, the fermentation group also showed a higher abundance of *Terrisporobacter* than the control group on day 1 (*P* = 0.016). However, on days 3 (*P* = 0.032) and 5 (*P* = 0.003) of storage, this abundance was higher in the control group. *Prevotella* abundance was comparable between the fermentation and control groups on day 1 (*P* = 0.066), but the former exhibited a higher *Prevotella* abundance than the latter on the 3rd (*P* = 0.034) and 5th (*P* = 0.046) days of storage (Fig. 8).

### Metabolomic analysis of fecal samples

**Fig 7:**
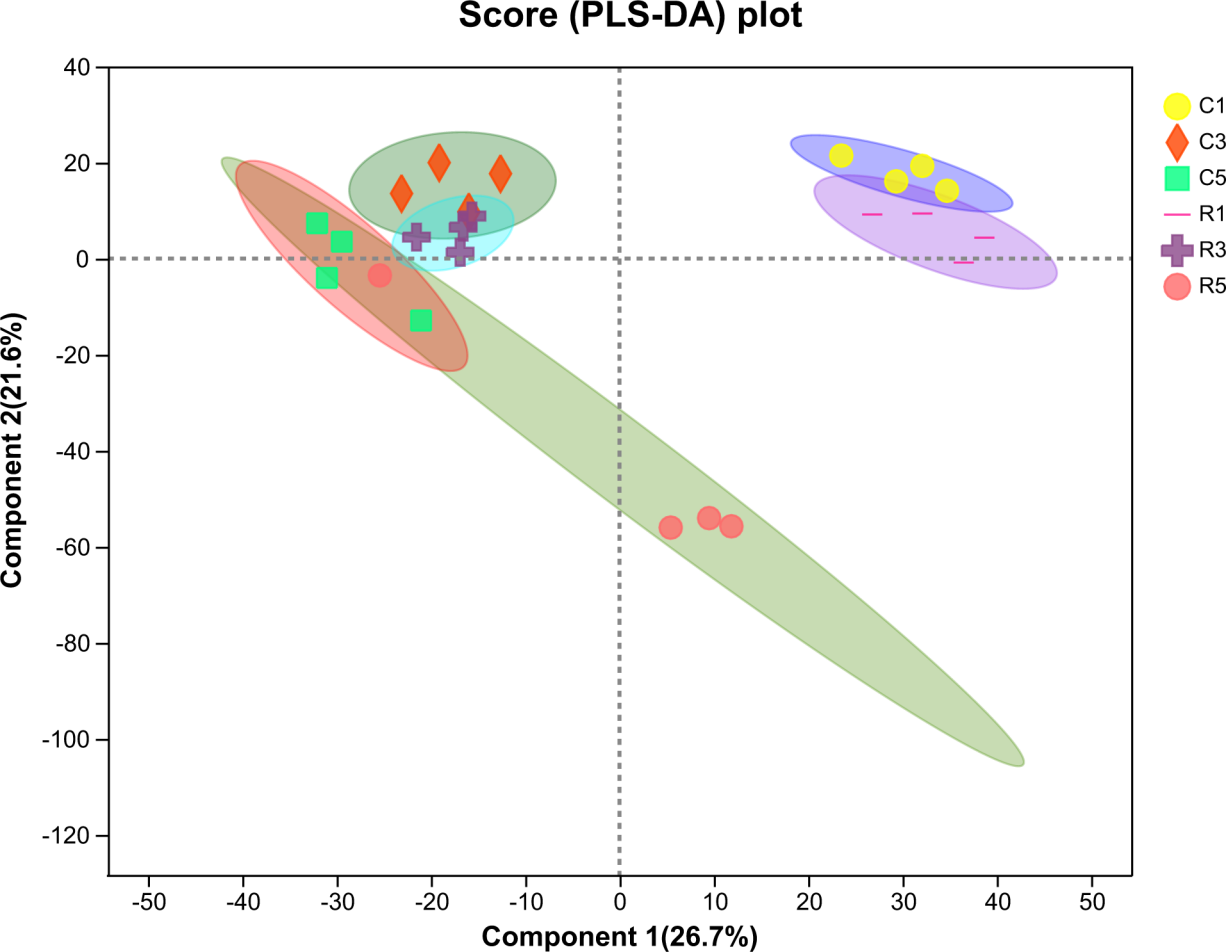
PLS-DA score plots of differential metabolites in fecal samples from growing pigs. C1: control group on the 1st day; C3: control group on the 3rd day; C5: control group on the 5th day; R1: fermentation group on the 1st day; R3: fermentation group on the 3rd day; and R5: fermentation group on the 5th day. *P < 0.05.

Figure 7 shows the score map obtained from PLS-DA for verifying between-group metabolomic differences. OPLS-DA highlights intergroup relationships according to simple visual examinations of spatial clustering patterns. In this study, the fecal samples from the two groups showed clearly distinct metabolomic profiles. The samples obtained on the 1st day were significantly separated from samples obtained on the 3rd and 5th days in both the fermentation and control groups.

### Differential metabolites among the different treatment groups

**Fig 8:**
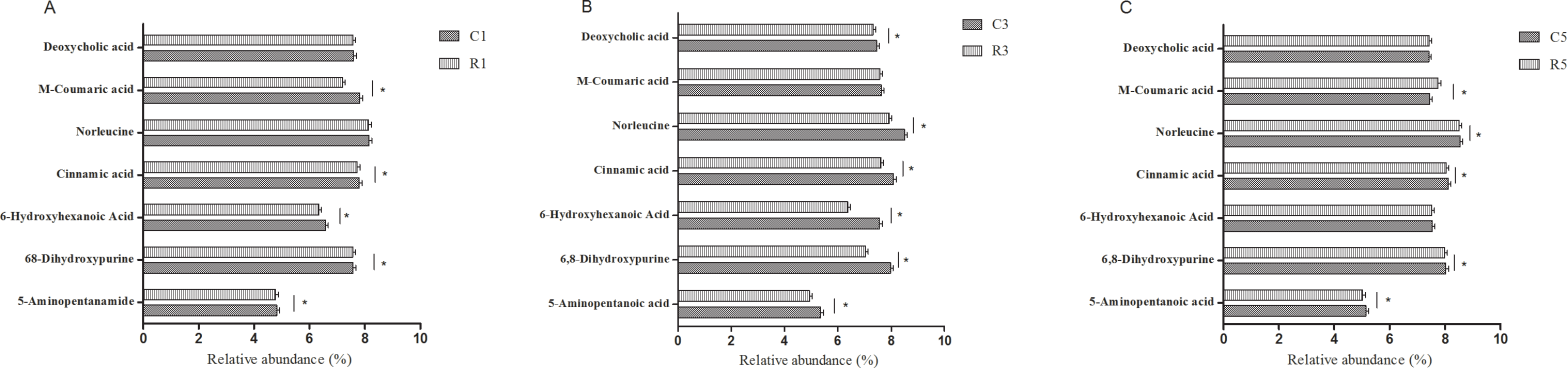
Analysis of major differential metabolites in fecal samples from growing pigs. (A) major differential metabolites on the 1^st^ day; (B) major differential metabolites on the 3^rd^ day; (C) major differential metabolites on the 5^th^ day. C1: control group on the 1st day; C3: control group on the 3rd day; C5: control group on the 5th day; R1: fermentation group on the 1st day; R3: fermentation group on the 3rd day; and R5: fermentation group on the 5th day. *P < 0.05.

The metabolite characteristics of the two groups of growing pigs are detailed in Table 3. Notably, 157 metabolites (65 positive ions and 92 negative ions), 29 metabolites (11 positive ions and 18 negative ions), and 686 metabolites (536 positive ions and 150 negative ions) showed differences between the fermentation and control groups on the 1st, 3rd, and 5th days of fecal storage. In particular, secondary amino acid metabolites, such as 6-hydroxyhexanoic acid, N-methyl-α-aminobutyric acid, 5-aminopentanoic acid, cinnamic acid, and norleucine, showed increasing levels with an increase in the fecal storage time in both groups. However, the rate of increase was slower in the fermentation group. In addition, in comparison to controls, fecal samples from the fermentation group exhibited a reduction in M-coumaric acid levels on the 1st day of fecal storage and a contrasting increase on the 3rd day (Fig. 8).

**TABLE 3:** Metabolite characteristics of fecal samples obtained from different groups of growing pigs^*a*^

Fermentation group vs control group	Metabolites	Positive ions	Negative ions	
1st	157	65	92	
3rd	29	11	18	
5th	686	536	150	

^*a*^ 1st, feces on the 1st day; 3rd, feces on the 3rd day; and 5th, feces on the 5th day.

### Differences in the enriched pathways

A total of 80, 118, and 179 Kyoto Encyclopedia of Genes and Genomes (KEGG) pathways were differentially enriched between fecal samples from the fermentation and control groups on days 1, 3, and 5 of storage. Most metabolites were linked to one or more metabolic pathways, microbial metabolism in diverse environments, the biosynthesis of secondary metabolites, and the biosynthesis of plant secondary metabolites with different levels of KEGG enrichment depending on the fecal storage period (Table 4).

**TABLE 4:** Major metabolic pathways detected in fecal samples from growing pigs^*a*^

Pathway name	R1 vs C1	R3 vs C3	R5 vs C5
Metabolic pathways	31	16	52
Biosynthesis of secondary metabolites	9	3	20
Microbial metabolism in diverse environments	8	4	12
Biosynthesis of plant secondary metabolites	7	2	6
Tryptophan metabolism	6	1	5
Biosynthesis of alkaloids derived from ornithine, lysine, and nicotinic acid	5	2	3
Tyrosine metabolism	4	1	3
Lysine degradation	4	3	4
Biosynthesis of alkaloids derived from shikimate pathway	3	2	5
Purine metabolism	1	2	3
Citrate cycle	3	0	1
Biosynthesis of amino acids	3	1	3

^*a*^ R1 vs C1, fermentation group vs control group on the 1st day; R3 vs C3, fermentation group vs control group on the 3rd day; and R5 vs C5, fermentation group vs control group on the 5th day.

### Isolation of *Weissella* and its inhibition of skatole *in vitro*

**Fig 9:**
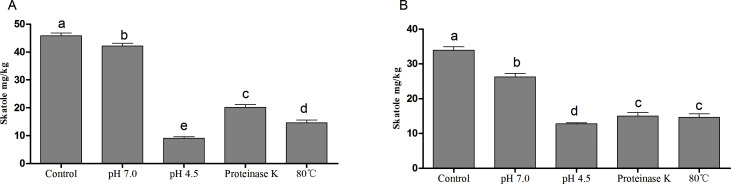
Effect of *Weissella cibaria* ZWC030 on skatole concentrations following different treatments: (A) 8 h and (B) 24 h. *P < 0.05.

The specific strain was identified as *Weissella cibaria* ZWC030 in our laboratory. The effect of the strain supernatant on the concentration of indole and skatole (Fig. 9) was examined. A higher skatole content was noted after treatment with the strain supernatant and pH adjustment to 7.0 than after treatment with protease K, at 80°C for both 8 h and 24 h. Meanwhile, treatment with the strain supernatant after adjustment to pH 4.5 yielded the lowest skatole content among all tested treatments.

## DISCUSSION

The interrelationships among intestinal health, the microbiota, and growth have attracted increasing attention in the pig farming industry (33). Several studies have reported that feeds supplemented with *Lactobacillus* spp. can enhance growth in pigs (34, 35). In line with these findings, the present study found that after 30 days of feed modification, pigs receiving fermented feed had higher final weights and ADG values as well as lower *F*/*G* ratios than pigs receiving standard feed. During fermentation, microorganisms convert basic substrates into new molecules, such as biomass, enzymes, and primary and secondary metabolites (36). *Lactobacillus* fermentation can yield compounds with biological activities and good bioavailability, including amino acids, peptides, and other nitrogenous compounds (37). Therefore, one possible explanation for our findings may be the increase in nutrient digestibility in pigs due to *Lactobacillus* fermentation.

The digestion and absorption of a majority of dietary proteins occur within the small intestine. Meanwhile, undigested proteins are transported to the hindgut, where they serve as substrates for microbial fermentation. In the present study, we used the nitrogen migration test to understand odor changes in pig feces. The results showed that the fermentation group had lower NH_4_^+^-N and higher NO_2_^−^-N values than the control group, with the conversion rates of NO_2_^−^-N/total N being 88.2% and 80.4%, respectively. These changes indicated that supplementation with feed fermented by *Lactobacillus* could reduce the environmental emissions of nitrogen from pig feces.

Results for the emission of odorous compounds (38–40), which are produced during the microbial degradation of nutrients (41–43), demonstrated that probiotics could decrease the contents of sulfur and ammonia compounds in feces, reducing their odor. The present study analyzed changes in fecal odor with prolonged storage of feces. The findings revealed that supplementation with feed fermented by *Lactobacillus* could reduce the emission of NH_3_, H_2_S, and CO_2_ from pig feces. The most obvious effect was the reduction in H_2_S emissions on the 3rd day. Meanwhile, NH_3_ and CO_2_ emissions were reduced by more than 23% and more than 22%, respectively. It had been said that the lactic acid produced by *Lactobacillus* strains could lower the pH in the digestive tract, thus increasing the activities of several important digestive enzymes (44). Growing pigs supplemented with multi-enzyme could significantly reduce fecal NH_3_ and H_2_S concentrations by more than 40% and 20% (45). In addition, the changes in gut microbiota caused by dietary supplementation could influence manure odor emissions. Hu et al. (46) reported that *Lactobacillus* promoted the growth of “good” bacteria and thereby reduced fecal ammonia concentrations. Therefore, the mechanism for *Lactobacillus-*fermented feed affecting the odor emission may be due to their functions in promoting the absorption of nutrients and improving the balance of the intestinal microbiota.

Indole and skatole are the two primary final products of intestinal bacteria. Among them, skatole is more easily noticeable as it is widespread in animal feces, wastewater, sewage sludges, etc., and its concentrations can reach 72.2 mg/kg (14, 47). Notably, its removal is very important (48). Successive studies have shown that *Rhodopseudomonas*, *Cupriavidus*, and *Acinetobacter* can remove skatole and have characterized the conditions for its degradation (49–52). Nevertheless, the use of these microbial strains for the degradation of skatole remains limited because skatole is biotoxic and recalcitrant. Our results suggest that skatole emissions can be reduced at their source by supplementing standard feed with *Lactobacillus* fermented feed in growing pigs.

The intestinal microbiota serves as a key player in the processes of nutrient digestion and absorption in pigs. Alterations in the gut microbiota due to dietary supplementation have been reported to influence odor emissions from manure (18, 53, 54). In our research, we first analyzed the alpha diversity of the microbiota during fecal storage, finding that the species richness significantly decreases as the storage time increases . Based on the diversity of the bacterial communities, the storage time significantly altered the relative abundance of certain taxa in the feces. The abundance of most microbes at the genus level decreased along with the time, for example, *Prevotella* and *Lachnospira* decreased on the 3rd day and disappeared on the 5th day. However, in our present study, feces in the fermentation group had higher community richness (ACE and Chao) and diversity (Simpson and Shannon index) than those in control group on the 3rd and 5th days, and the genus of *Weissella*, *Pediococcus*, *Bacteroides*, and *Acinetobacter* in the control group appeared to be diminished compared with fermentation group. A study conducted by Yang et al. (55) revealed that diet supplementation with soybean oligosaccharides increases the richness of the microbiota and decreases skatole levels in the cecal digesta of broilers. Liu et al. (22) reported that the alpha diversity of the microbiota in broilers was the highest, and the concentration of skatole in cecal digesta was the lowest, after supplementation with 3.5% soybean oligosaccharides than after supplementation at levels of 0.0%, 0.5%, and 2.0%. Our study, therefore, also demonstrated a correlation between the odor concentration and microbiota diversity.

We used PLS-DA to analyze the fecal microbiota differences at the OTU level between the two treatment conditions, and dots of different colors or shapes represented fecal samples under different conditions. In the present results, owing to the prolongation of fecal storage, the microbial composition on day 5 was more different from that on day 1 in control samples (*x*-axis). The results showed that significant differences were found between the fecal samples in fermentation and control groups (*y*-axis). The PLS-DA was consistent with the distribution results obtained from microbiota. As the storage time increased, the abundance of Bacteroidetes phylum significantly decreased. However, pigs in fermentation group exhibited a higher abundance of Bacteroidetes. In addition, the fermentation group exhibited a higher *Prevotella* abundance on the 3rd and 5th days of fecal storage. *Prevotella* has been reported to dominate the fecal metagenome of swine animals and is crucial for carbohydrate metabolism (56). Therefore, we speculate that there is a close relationship between the abundance of Bacteroidetes, including *Prevotella*, and the emission of odors. Moreover, our present study showed an increase in the abundance of *Streptococcus* with increasing storage time, and the fermentation group showed a lower abundance of *Streptococcus*. In the large intestine of monogastric animals, the proteolytic activity has been mainly attributed to the genera of *Streptococcus*, *Propionibacterium*, *Fusobacterium*, and *Clostridium* (11), and a high abundance of *Streptococcus* is considered pathogenic in swine (57). Zhou et al. (58) reported that odor could be reduced through inhibiting the growth of pathogenic microbes. Liu et al. (22) suggested that odor production was associated with the abundance of *Escherichia* genus. Our earlier studies indicated that the addition of *Lactobacillus reuteri* ZLR003 to weaned piglets (59) and *L. acidophilus* to growing pigs (60) could decrease the abundance of fecal *Streptococcus*. All of these results suggested that the odor reduction in our present study might be associated with the decreased abundance of *Streptococcus*.

Our present study also suggests that *L. acidophilus* can regulate the proportion of the genus *Weissella* in feces. It has been said that the *Weissella* genus is well known for producing several bacteriocins, known as weissellicins, which represent a potential biotechnological tool for killing pathogens and maintaining biopreservation (61–63). *Weissella* species are commonly used in traditional fermentation (64), but reports on their use in animal production have been limited. A previous study found that fermenting Sichuan pickle with *Weissella cibaria* and *Lactobacillus plantarum* could improve its quality; in particular, the pH value of Sichuan pickle decreased from 7.88–7.58 to 3.55–3.45 when compared with Sichuan pickle fermented using mono-inoculation with *Lactobacillus plantarum*. Moreover, four additional non-volatile organic acids (lactic acid, acetic acid, malic acid, and citric acid) could be detected (65). The results remind us that the increased abundance of *Weisseria* induced by *Lactobacillus acidophilus* fermentation feed may promote the utilization of amino acids and reduce the production of odor, but further research is still warranted.

Few studies have reported metabolite changes during fecal storage. In the present study, the contents of secondary amino acid metabolites, such as 6-hydroxyhexanoic acid, N-methyl-α-aminobutyric acid, 5-aminopentanoic acid, cinnamic acid, and norleucine, increased as the storage time increased, irrespective of which diet the growing pigs were on. However, the rate of increase was slower in the fermentation group. The production of 5-aminopentanoic acid is related to amino acid metabolism in organisms, with a previous study reporting that its contents significantly increase in frogs exposed to Pb pulses (66). Metabolites altered by 5-aminopentanoic acid have also been identified in the plasma of patients with angioimmunoblastic T-cell lymphoma (54). However, in our study, the contents of M-coumaric acid first decreased and then increased in the fermentation group. M-coumaric acid is an isomer of hydroxycinnamic acid, with p-coumaric acid exerting an inhibitory effect on tyrosinase and showing some beneficial effects, such as the suppression of IL-8 and the subsequent enhancement of antioxidant enzyme activity (67). Overall, fresh feces have an abundance of microbiota and metabolites, which serve as key players in the processes of nutrient digestion and absorption in pigs. However, previous research showed that urine is used to measure patterns of metabolic components, with total energy and nitrogen being the most common components estimated (68). Therefore, we may consider using a fecal-urine mixture for further research.

Previously, some Gram-negative and aerobic bacterial strains, such as *Pseudomonas aeruginosa* Gs (69), *Pseudomonas putida* LPC24 (70), and *Burkholderia* sp. IDO3 (71), had been isolated with skatole-degrading capacity. One key innovation of our present study was the isolation of *Weissella cibaria* ZWC030, which was derived from pig intestines and belonged to facultative anaerobic bacteria. We found that the skatole content was higher after treatment with this strain’s supernatant and adjustment to pH 7.0 than after treatment with protease K, at 80°C for 8 h and 24 h. However, when the supernatant pH was adjusted to 4.5, it yielded the lowest skatole concentration among all the treatment groups. It has been reported that skatole is produced from indoleacetic acid via decarboxylation, which is catalyzed by indoleacetic acid decarboxylase (14)—a pH-sensitive member of the glycine radical enzyme superfamily (72). Therefore, our preliminary skatole inhibition test indicated that the organic acids produced by *Weissella cibaria* ZWC030 could inhibit the conversion of tryptophan to skatole, which showed good characteristic in the reducing of fecal odors.

### Conclusion

Overall, this study showed that dietary supplementation with feed fermented by *Lactobacillus* could regulate the microbiota and inhibit the growth of harmful bacteria. Additionally, it could prevent the accumulation of toxic substances and reduce odor emission from pig feces, thereby reducing environmental pollution. Although rigorous future studies are warranted in this field to address some unanswered questions, our findings provide a novel avenue for the research and development of special probiotics that promote reduced odor emission from pig feces.

## Data Availability

Data have been deposited in BioProject under accession number PRJNA956336.
